# Impact of the censoring distribution on time-to-event problems in the presence of competing risks

**DOI:** 10.1186/1745-6215-12-S1-A140

**Published:** 2011-12-13

**Authors:** Mark W Donoghoe, Val Gebski

**Affiliations:** 1 NHMRC Clinical Trials Centre, University of Sydney, Sydney, New South Wales, Australia

## Objectives

Methods accounting for competing risks in time-to-event problems are becoming common in mainstream statistical analyses. Standard approaches include those based on log-rank type tests [1] and cumulative incidence regression [2]. These approaches are based on weighting competing events by the censoring distribution. The usual cumulative incidence regression uses weights based on the pooled censoring distribution. However, the impact of the pattern of events and censoring in these approaches is still unclear. We examine two aspects of this problem: the amount of competing risk present in a proportional hazards model, and the pattern of censoring between groups in the presence of competing risks.

## Methods

We investigated the behaviour of the estimate of treatment effect under different (i) rates of competing risk events and (ii) censoring patterns. Through extensive simulations, we compared event-specific analyses to competing risk approaches, and also the power and type I error of different weighting schemes for common methods of analysis under these conditions. These approaches were also examined on actual data on time to relapse in patients receiving bone marrow transplant for multiple myeloma, with the competing risk being transplant-related mortality.

## Results

Differences between treatment effect estimates using proportional hazards and proportional subhazards methods were evident at competing event rates as low as 2.5%, even when there was no difference in competing risks between treatment groups. For the multiple myeloma example, the p-values were 0.0003 (log-rank), 0.005 [1] and 0.051 [2]. When censoring was evenly distributed between groups, the different weighting approaches yielded similar results. However, when there was differential censoring between the groups, the usual cumulative incidence regression produced inflated Type I errors (exceeding 10% when the hazard ratio for censoring was greater than 2) and reduced power when compared to a method where weights were calculated separately within treatment groups.

## Conclusions

Competing risk approaches should be included in time-to-event analyses, even when the rate of competing events is expected to be low. When comparing two groups, using weights from separate censoring distributions is recommended as this has a less inflated Type I error and greater power. This may be thought of as analogous to using separate variances when comparing two means rather than using a pooled variance, although the increased efficiency of the pooled approach does not exist here when there is no true difference in censoring risk.
